# Therapeutic Effects of Traditional Chinese Medicine on Spinal Cord Injury: A Promising Supplementary Treatment in Future

**DOI:** 10.1155/2016/8958721

**Published:** 2016-03-28

**Authors:** Qian Zhang, Hao Yang, Jing An, Rui Zhang, Bo Chen, Ding-Jun Hao

**Affiliations:** ^1^Translational Medicine Center, Hong Hui Hospital, Xi'an Jiaotong University College of Medicine, Xi'an 710054, China; ^2^Department of Spine Surgery, Hong Hui Hospital, Xi'an Jiaotong University College of Medicine, Xi'an, China

## Abstract

*Objective*. Spinal cord injury (SCI) is a devastating neurological disorder caused by trauma. Pathophysiological events occurring after SCI include acute, subacute, and chronic phases, while complex mechanisms are comprised. As an abundant source of natural drugs, Traditional Chinese Medicine (TCM) attracts much attention in SCI treatment recently. Hence, this review provides an overview of pathophysiology of SCI and TCM application in its therapy.* Methods*. Information was collected from articles published in peer-reviewed journals via electronic search (PubMed, SciFinder, Google Scholar, Web of Science, and CNKI), as well as from master's dissertations, doctoral dissertations, and Chinese Pharmacopoeia.* Results*. Both active ingredients and herbs could exert prevention and treatment against SCI, which is linked to antioxidant, anti-inflammatory, neuroprotective, or antiapoptosis effects. The detailed information of six active natural ingredients (i.e., curcumin, resveratrol, epigallocatechin gallate, ligustrazine, quercitrin, and puerarin) and five commonly used herbs (i.e., Danshen, Ginkgo, Ginseng, Notoginseng, and Astragali Radix) was elucidated and summarized.* Conclusions*. As an important supplementary treatment, TCM may provide benefits in repair of injured spinal cord. With a general consensus that future clinical approaches will be diversified and a combination of multiple strategies, TCM is likely to attract greater attention in SCI treatment.

## 1. Introduction

Spinal cord injury (SCI) is a catastrophic event that can profoundly affect a patient's life, with far-reaching social and economic effects. The estimated annual global incidence of SCI is approximately 15–40 cases per million and is increasing with the development of modern society [1]. Thus, the treatment of SCI is currently a significant challenge in clinic and in research around the world. With ongoing advances in neurobiology, materials science, pharmacology, and other related sciences, great progress has been made in the prevention and treatment of SCI. Considerable advances have been made to relieve the symptoms of SCI and the suffering of patients, which are achieved by preventing injury progression, managing deafferentation pain syndromes, implementing bowel and bladder training regimens, and teaching patients to cope with their disabilities [2].

Today, the routine therapy employed in the early stage of SCI mainly involves surgical procedures combined with high-dose methylprednisolone (MP). The surgical procedures might stabilize and decompress spinal cord, while MP can inhibit lipid peroxidation, maintain the blood-spinal cord barrier, enhance spinal cord blow flow, inhibit endorphin release, and limit the inflammatory response. However, the question of optimal timing of surgical interventions (or even the intervention itself) has generated considerable debate and remains unanswered. Moreover, MP is also highly controversial due to a lack of consensus with regard to its true beneficial effects [3, 4]. Thus, various other novel strategies for SCI repair have emerged and received considerable research focus, including cell therapy (e.g., transplantation of neural stem cells, mesenchymal stem cells, olfactory ensheathing cells, Schwann cells, activated macrophages, and embryonic stem cells), molecular therapy (e.g., neurotrophin, anti-Nogo antibody, and interleukin-10), and tissue engineering (e.g., construction of a 3D scaffold including hydrogels, sponges, guidance tubes, and nanofibrous scaffolds) [2]. However, more related research needs to be carried out before these novel strategies are widely applied in clinic.

As an abundant source of natural drugs, Traditional Chinese Medicine (TCM) has many thousands of years of history in clinical applications in China and other Asian countries. TCM comprises hundreds of commonly used herbs, which is mostly used in intervention therapy as a form of compound prescription in clinic. Each herb contains numerous chemical constituents which belong to different categories, and the active ingredients exert therapeutic action in the treatment of some disease. In recent years, TCM has attracted much attention in the field of SCI treatment. Both active ingredients and herbs [5–8], and even compound prescriptions [9–12], have shown effectiveness in the prevention and treatment of SCI. Although it could not replace regular surgical procedures, as a complementary and alternative treatment, intervention of TCM plays an important role in preoperative prevention and postoperative recovery. Meanwhile, combined application of multiple therapeutic approaches would benefit the functional recovery of spinal cord, for example, TCM combined with cell therapy, molecular therapy, or tissue engineering. Thus, application of TCM would be an important focus of future research in the field of SCI treatment.

This review intends to provide an overview of TCM applications in the field of SCI. It will provide the readers with the detailed prevention and treatment effects of six active natural ingredients (i.e., curcumin, resveratrol, epigallocatechin gallate, ligustrazine, quercitrin, and puerarin) and five commonly used herbs (i.e., Danshen, Ginkgo, Ginseng, Notoginseng, and Astragali Radix), on the basis of the pathophysiology and therapeutic mechanisms of SCI.

## 2. Pathophysiology of SCI

In the past 30–40 years, much research has focused on elucidating the mechanisms and complex pathophysiologic processes of SCI. Pathophysiological events occurring after SCI include acute, subacute, and chronic phases.

The acute phase refers to the immediate postinjury period, when the spinal cord is lacerated or macerated by a sharp penetrating force, contused or compressed by a blunt force (most common), or infarcted by a vascular insult [13]. This stage is also called “primary injury,” and the processes cannot be reversed. The extent of injury is hard to control, which depends on the violence severity, compression time of spinal cord, fracture-dislocation situation of spine, and acceleration of impact force, as well as absorption circumstances of impact energy by surrounding tissues.

The subacute phase occurs over the time course of minutes to weeks following SCI and leads to further damage that is described as “secondary injury.” The secondary stage consists of the following events. (1) Vascular changes: it includes hemorrhage, thrombosis, vasospasm, loss of autoregulation, breakdown of blood brain barrier, and infiltration of inflammatory cells. This leads to edema, necrosis, and ischemia [14]. (2) Free radical formation and lipid peroxidation: SCI results in a rapid and extensive oxidative stress reaction, which causes oxidative death of the spinal cord neurons and reduces spinal cord blood flow that leads to edema and an inflammatory response [15]. (3) Disruption of an ionic balance of K^+^, Na^+^, and Ca^2+^: it leads to depolarization of cell membranes, ATPase failure, and increase of intracellular Ca^2+^. (4) Glutamate excitotoxicity: it is an increased release of extracellular glutamate after SCI that induces excessive activation of glutamate receptors leading to further neuronal cell death. (5) Apoptosis: it is a form of programmed cell death seen in populations of neurons, oligodendrocytes, microglia, and, perhaps, astrocytes after SCI [16]. The death of oligodendrocytes in white matter tracts continues for many weeks after injury and may contribute to postinjury demyelination [17]. (6) Inflammatory response: resident microglia are activated after SCI, along with proinflammatory cytokines that are produced by infiltrating neutrophils and macrophages, which induce higher extravasation of leukocytes and further tissue damage of the surrounding original injury site [18]. Nevertheless, some studies have demonstrated that inflammation also plays an important role in neural tissue repair [19].

Finally, the chronic phase of SCI occurs days to even years after injury and comprises many events, such as white matter demyelination, gray matter dissolution, connective tissue deposition, and reactive gliosis, that lead to glial scar formation. Microglia and astrocytes become activated, undergo proliferation, increase in size, activate astrogliosis, and produce a glial scar, which subsequently inhibits regeneration of neurons. Increased glial fibrillary acidic protein (GFAP) expression is a hallmark of reactive astrocytes, and this cytoskeletal protein contributes to the barrier effect [20]. A cystic cavity is surrounded by glial scar that progressively expands and leads to a condition called syringomyelia in approximately 25% of SCI patients. Finally, in many cases, SCI leads to neurological impairments in both orthograde and retrograde directions, including brain regions, as well as the development of pain syndromes and mood disorders such as depression [21].

## 3. Pharmacological Intervention with Natural Compounds

### 3.1. Curcumin

**Figure 1:**
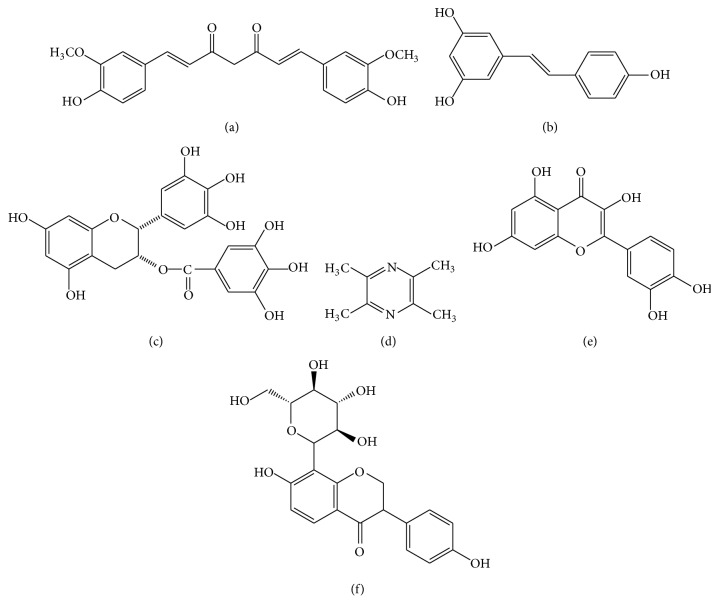
Structures of natural compounds ((a) curcumin; (b) resveratrol; (c) epigallocatechin gallate (EGCG); (d) ligustrazine; (e) quercitrin; (f) puerarin).

Curcumin is a natural polyphenolic compound extracted from Curcumae Longae Rhizoma, the dried rhizome of* Curcuma longa *L. in Zingiberaceae [22], which is prevalent in tropical and subtropical regions including India, China, and Southeast Asia. Structure of curcumin is shown in Figure 1(a), and studies indicate that it has potent anticancer, antiarthritic, and antidiabetic activities [23].

In recent years, curcumin has emerged as a potential therapeutic drug in SCI treatment. Behavioral scores, including tilt board test and Basso, Beattie, and Bresnahan (BBB) scores, have confirmed improvement in rat hindlimb motor functions (*P* < 0.01) [24, 25]. Wet/dry weight ratio assay showed that administration of curcumin (100 mg/kg, i.p.) could significantly alleviate edema of the injured spinal cord (*P* < 0.01) [26]. Many studies have indicated that curcumin exerts a treatment effect in SCI by protecting neurons, inhibiting oxidant and inflammatory reactions.

#### 3.1.1. Antioxidant and Anti-Inflammatory Effects

The antioxidant and anti-inflammatory effects of curcumin have been demonstrated by many studies [27]; thus it is reasonable that curcumin is used in SCI treatment. In the group receiving curcumin (200 mg/kg/d, p.o.), serum superoxide dismutase (SOD) level was significantly increased (*P* < 0.01), while the malondialdehyde (MDA) level significantly decreased (*P* < 0.05) compared with the control group and MP group [28]. As an important measure of oxidative stress, glutathione (GSH) level, glutathione/oxidized-glutathione ratio (GSH/GSSH), and glutathione peroxidase (GSH-PX) level all increased, while catalase (CAT) level decreased significantly* in vitro* (SK-N-SH cells) and* in vivo* (SCI mice) [24, 29].

In the context of anti-inflammatory pathways, inflammatory cytokines network is believed to be central to the pathophysiology of inflammatory processes. After SCI, the inflammatory related factors including NF-*κ*B and proinflammatory cytokines (e.g., TNF-*α*, IL-1*β*, IL-6, and RANTES) in the injured spinal cord were significantly upregulated following SCI and could be suppressed when treated with curcumin (100 mg/kg, i.p.) [26, 30]. In the antioxidant Nrf2/ARE pathway, which regulates the expression of inflammatory cytokines, the activity of pleiotropic transcription factor Nrf2 was significantly activated following SCI and could be further induced when treated with curcumin. Induction of Nrf2 activity by curcumin markedly decreased NF-*κ*B activation and reduced expression of TNF-*α* and IL-1*β* [26].

#### 3.1.2. Neuroprotective and Antiapoptosis Effects

As a hallmark of reactive astrocytes, increased GFAP expression indicates the production of glial scar and inhibition of axonal extension. After treatment with curcumin (1 *μ*M) for 7 d, GFAP mRNA isolated from reactivated astrocytes was significantly downregulated (*P* < 0.001), and a reduction of GFAP protein expression was observed following immunofluorescent staining of primary cultured astrocytes* in vitro* [25]. Western immunoblotting signals showed that level of neuron-specific enolase (NSE) was significantly increased in curcumin posttreatment groups (1 *μ*M, 24 h), indicating protection and preservation of the neuronal phenotype [29]. As the key intracellular cysteine protease of the cascade of events associated with apoptosis, both caspase-3 and caspase-7 were activated and significantly upregulated after neural injury. Administration of curcumin significantly and dose-dependently inhibited caspase-3 and caspase-7 levels and exerted antiapoptosis effects [29, 31, 32].

### 3.2. Resveratrol

Resveratrol (Figure 1(b)) is a natural polyphenol antioxidant and the active constituent of* Polygonum cuspidatum* (Japanese knotweed, 0.524 mg/g), red wine (0.1–14.3 mg/L), red grape skins (50–100 *μ*g/g), and berries such as blueberries, as well as peanuts and other nuts [33]. As early as 1970, it was noticed that there was a positive relationship between wine consumption and the incidence of heart disease [34]. Through modern pharmacological research, resveratrol has been widely used in preventing or slowing the progression of a wide variety of diseases, including cardiovascular disease, cancer, ischemic injury, and Alzheimer's disease [33, 35].

In recent years, the potential therapeutic effect of resveratrol in SCI treatment has been confirmed by behavioral scores (i.e., BBB scores, Rivlin, and Tator's angle board test) and histopathological changes [36, 37]. Hematoxylin and eosin (HE) staining showed that administration of resveratrol (100 mg/kg or 200 mg/kg, i.p.) could alleviate hemorrhage and edema in both gray and white matter as well as reversing the tissue necrosis, liquefaction, pyknosis, and dissolution of nucleus, and appearance of apoptotic bodies 3 d after SCI. Nissl staining showed that, with resveratrol intervention, neurons displayed partially restored functions, especially in cellular nutrient supply and energy biosynthesis [38]. According to previous reports, resveratrol protects the injured spinal cord mainly by inhibiting oxidant formation and apoptosis.

#### 3.2.1. Antioxidant Effects

Many studies have shown the effectiveness of resveratrol on oxidative stress caused by SCI. In an SCI model, resveratrol (100 *μ*g/kg or 10 mg/kg, i.v.; 50 mg/kg or 100 mg/kg, i.p.) reduced the expression and level of MDA in injured spinal cord tissues (*P* < 0.05) [36, 37, 39, 40]. Additionally, the increase of xanthine oxidase (XO) and NO levels, as well as the decrease of SOD activity and GSH level in rat injured spinal cord tissues, could be reversed (*P* < 0.01) by administration of resveratrol after SCI (100 mg/kg or 200 mg/kg, i.p.) [36, 38].

#### 3.2.2. Antiapoptosis Effects

Firstly, electron microscopic (EM) identification and TUNEL staining clearly showed that the number of TUNEL-positive cells distributed dramatically decreased after resveratrol treatment in both the white and the gray matter of spinal cord in an SCI rat model (*P* < 0.01). By contrast, the apoptosis index significantly declined (*P* < 0.01). Resveratrol (200 mg/kg, i.p.) could significantly improve abnormal morphology, including neuronal shrinking, breaking, or disappearance of mitochondrial ridges, cytoplasm vacuolization, and marked enlarging of the endoplasmic reticulum [38]. Secondly, resveratrol intervention obviously inhibited the upregulation of protein expression of the proapoptosis factor Bax and the terminal executing enzyme for substrate cleavage of caspase-3 (*P* < 0.01). In addition, resveratrol inhibited the downregulation in the protein expression of the antiapoptosis factor Bcl-2 (*P* < 0.01) that was induced by SCI [38]. Finally, several studies have revealed that resveratrol (20 mg/kg or 60 mg/kg, i.p.) could activate the PI3K/Akt pathway, prevent neuronal apoptosis [41], and attenuate activation of the mitogen-activated protein kinases (MAPKs) signaling pathways [42].

### 3.3. Epigallocatechin Gallate (EGCG)

The natural product (–)-epigallocatechin-3-gallate (EGCG, Figure 1(c)) is the major polyphenolic constituent found in green tea (dried fresh leaves of the plant* Camellia sinensis* L. Ktze. in Theaceae) [43]. As the second most consumed beverage globally, numerous epidemiological studies have reported an inverse association between tea consumption and cardiovascular events. Moreover, ingestion of green teas significantly increased anticarcinogenic, anti-inflammatory, antioxidant, antithrombotic, and antimutagenic capacity. A number of scientific researches have suggested that EGCG is responsible for the majority of the potential health benefits attributed to green tea consumption [44].

In recent years, research has focused on the therapeutic effect of EGCG in SCI that is attributed to its potent antioxidant, anti-inflammatory, antiapoptotic, and neuroprotective activities [45]. After administration of EGCG (10 mg/kg or 20 mg/kg, i.p.) for 1 w after SCI, behavioral scores (i.e., BBB scores and tilt board test) have confirmed an improvement of rat locomotor functional recovery (*P* < 0.05). LFB staining indicated that EGCG administration decreased the cavity area, while increasing the myelin sheath area as compared with the SCI group [46, 47].

#### 3.3.1. Antioxidant and Anti-Inflammatory Effects

The study of Deng et al. indicated that EGCG (50 mg/kg or 100 mg/kg, i.p.) could significantly upregulate the levels of O^2-^ and SOD, while significantly downregulating the activity of MDA 24 h following SCI (*P* < 0.01). Meanwhile, EGCG (50 mg/kg or 100 mg/kg, i.p.) significantly reduced the production of IL-1*β*, TNF-*α*, and ICAM-1 in SCI rat serum (*P* < 0.01). In particular, the antioxidant and anti-inflammatory effects of 100 mg/kg EGCG were similar to MP in the treatment of SCI (*P* > 0.05) [48, 49].

#### 3.3.2. Antiapoptosis and Neuroprotective Effects

The level of Bcl-2 was depressed, while Bax level and the Bcl-2/Bax ratio were increased in spinal cord tissues 24 h after SCl (*P* < 0.01). EGCG (50 or 100 mg/kg, i.p.) treatment significantly increased Bcl-2 level, and the Bcl-2/Bax ratio, while decreasing Bax level [48].

EGCG enhanced the expression of endogenous neurotrophic factors like neurotrophin-3 (NT-3) and brain-derived neurotrophic factor (BDNF) in SCI rats and protected spinal motor neurons from death after SCI [48]. In the study of Ge et al., both IHC and Western blot assays showed that aquaporin-4 (AQP4) and GFAP expression were significantly increased from 24 to 72 h after SCI, while EGCG treatment (100 mg/kg, i.p.) obviously decreased its expression. Conceivably, the downregulation of AQP4 expression by EGCG (100 mg/kg, i.p.) treatment might be beneficial to reducing spinal cord edema in SCI rats [50].

### 3.4. Ligustrazine

Ligustrazine (tetramethylpyrazine, TMP, Figure 1(d)) is a natural alkaloid extracted from Chuanxiong Rhizoma, the dried rhizome of* Ligusticum chuanxiong* Hort. in Umbelliferae, which is chiefly found in China [51]. In TCM, Chuanxiong Rhizoma can treat neurovascular, cardiovascular, and brain and kidney diseases, while TMP has a diverse array of pharmacological functions, including dilation of blood vessels, inhibition of platelet aggregation, improvement of microcirculation, inhibition of cell apoptosis, elimination of oxygen free radicals, and exertion of a calcium antagonist action [52, 53].

In recent years, it has been revealed that TMP could protect injured spinal cord by suppressing inflammatory cytokines, inhibiting cell apoptosis, and scavenging oxygen free radicals. TMP (30 mg/kg, i.p.) could significantly increase behavioral scores (i.e., BBB scores) and improve rat hindlimb motor functions (*P* < 0.01) [54].

#### 3.4.1. Anti-Inflammatory and Antioxidant Effects

Systemic administration of TMP (30 mg/kg, i.p.) exerted potent neuroprotective effects against spinal cord injury by reducing the expression of proinflammatory cytokines (i.e., IL-1*β* and TNF-*α*), upregulating the expression of anti-inflammatory cytokine IL-10, and inhibiting NF-*κ*B activation (*P* < 0.01) [54]. Several studies suggested that TMP effectively protects the central nervous system by scavenging reactive oxygen species and regulating nitric oxide production, and consequently preventing peroxynitrite formation [52]. SCI significantly decreased SOD level and increased MDA level in spinal cord as compared with the sham group (*P* < 0.05). In addition, TMP treatment (30 mg/kg, i.p.) significantly reversed the changes in SOD and MDA activities (*P* < 0.01) [55], while significantly suppressing oxidative stress and preventing excitotoxic cell damage in neuronal cultures [56].

#### 3.4.2. Antiapoptosis Effects

Western blot analysis showed that spinal cord injury obviously reduced Bcl-2 expression and increased Bax expression as compared with the sham group (*P* < 0.01), while treatment with TMP (30 mg/kg, i.p.) was associated with greater Bcl-2 and attenuated Bax expression relative to the vehicle control group (*P* < 0.01) [55]. TMP could also reduce caspase-3 activity, activate the PI3K/Akt pathway, inhibit neuronal apoptosis, and prevent neuronal loss [57, 58].

### 3.5. Quercitrin

Quercetin (Figure 1(e)) is a typical flavonol-type flavonoid that is ubiquitously present in many fruits and vegetables, such as apples, onions, citrus fruits, berries, red grapes, red wine, and broccoli. As a flavonol essential in many plants, quercetin is rich mainly in its sugar derivatives [59]. Quercetin exhibits antioxidative, anti-inflammatory, and vasodilating activity and has been proposed as a potential approach in the prevention and therapy of cardiovascular diseases and cancer. Recently, quercetin has been marketed in the United States primarily as a dietary supplement [60, 61].

Recently, quercetin has emerged as a potential therapeutic drug in the treatment of SCI. Quercetin could significantly increase BBB scores and inclined plane test score in SCI rats, which is similar to the positive control drug MP [62]. Many studies have indicated that SCI treatment of quercetin is attributed to its antioxidant, anti-inflammatory, and antiapoptosis activities.

#### 3.5.1. Antioxidant and Anti-Inflammatory Effects

Quercetin treatment (20 mg/kg twice daily, i.p.) could reverse the upregulation of MDA level (*P* < 0.05), NO level (*P* < 0.001), and MPO activity (*P* < 0.001) and the downregulation of GSH level (*P* < 0.001) and SOD activities (*P* < 0.05) after SCI, thus reducing oxidative damage in tissues [63]. Immunohistochemistry results also showed that the rate of iNOS-positive cells was significantly higher from days 1 to 7 postoperatively (*P* < 0.05), while being significantly lower in the injured spinal cord after administration of quercetin (0.2 mg/kg/d, i.p.) (*P* < 0.05) [62].

Enzyme-linked immunosorbent assay (ELISA) showed that plasma TNF-*α*, IL-1*β*, and IL-6 levels were significantly increased in the vehicle-treated SCI group (*P* < 0.01–*P* < 0.001), whereas treatment with quercetin suppressed any increases of these proinflammatory cytokines (*P* < 0.05–*P* < 0.001) [63].

#### 3.5.2. Antiapoptosis Effects

There was no statistically significant difference between the quercetin and p38MAPK inhibitor (SB203580) treatment groups (*P* > 0.05), which indicated that the potential mechanism of action of quercetin is through inhibiting the activation of the p38MAPK signaling pathway [62]. Moreover, semiquantitative Western blot analysis revealed that increased caspase-3 protein expression in bladder tissues of SCI rats was attenuated following quercetin treatment (0.2 mg/kg/d, i.p.) (*P* < 0.01) [63].

### 3.6. Puerarin

Puerarin (Figure 1(f)) is the most important phytoestrogen extracted from the dried root of* Pueraria lobata* (Willd.) Ohwi in Leguminosae, which is a commonly used traditional Chinese medicine. Researchers have concentrated on the pharmacological activities of puerarin, which displays a series of beneficial activities on hangover, cardiovascular disease, osteoporosis, neurological dysfunction, fever, and liver injury in clinical treatment and experimental research [64].

In recent years, several studies have shown that puerarin was effective in treating SCI. Administration of puerarin (50 mg/kg/d, i.p.) for 3 d significantly improved motor function (neurological deficit score 48 h after SCI, *P* < 0.05) and reduced spinal infarction volume (*P* < 0.05), while the optimal time of treatment with puerarin was within 4 h after SCI [65]. The therapeutic effect of puerarin on SCI was mainly attributed to its neuroprotective activity.

Puerarin exhibits a neuroprotective action in SCI and is associated with several aspects. Firstly, following SCI, the expression of p35 was downregulated, while p25 was upregulated in a mechanism dependent on cleaving p35. The enhanced expression of p25 resulted in a hyperactivation of Cdk5. The pretreatment with puerarin significantly depressed the upregulation of p25 and inhibited the downregulation of p35 (*P* < 0.05), by way of a roscovitine-like function [66]. Secondly, it is well-known that release of a high amount of glutamate and activation of metabotropic glutamate receptors lead to spinal tissue injury following SCI. The excitotoxicity of glutamate to spinal cells is mediated via glutamate receptors of the spinal cord. Intraperitoneal injection with puerarin (50 mg/kg), at 1 h, 2 h, 4 h, and 6 h after SCI, significantly decreased glutamate release (*P* < 0.05) and inhibited mGluR mRNA expression (*P* < 0.05) [67]. Thirdly, puerarin treatment significantly reversed the decrease in Trx-1 and Trx-2 mRNA expression after SCI (*P* < 0.05) and elevated number of apoptotic cells in the spinal cord (*P* = 0.01) [65].

### 3.7. Summary

The sources, structures, doses, and mechanisms of all six natural compounds in SCI treatment are summarized in Table 1. Besides the six natural compounds mentioned above, there are also some other compounds that have been reported in the treatment of SCI, including hydroxysafflor yellow A [68], tetrandrine [69], and piperine [70]. However, there are insufficient studies describing SCI treatment of these compounds, and more related research is required before these compounds are considered as potential therapeutic agents in treating SCI.

## 4. Pharmacological Intervention with Chinese Herbs

### 4.1. Danshen

#### 4.1.1. Source, Chemical Constituents, and Pharmacology of Danshen

Danshen (Salviae Miltiorrhizae Radix et Rhizoma) is the dried root and rhizome of* Salvia miltiorrhiza* Bge. in genus* Salvia* of mint family [22]. As one of the best-known Chinese traditional herbs, it has been clinically used for more than 2000 years and is mainly produced in Anhui, Shanxi, Hebei, and Jiangsu provinces in China.

**Figure 2:**
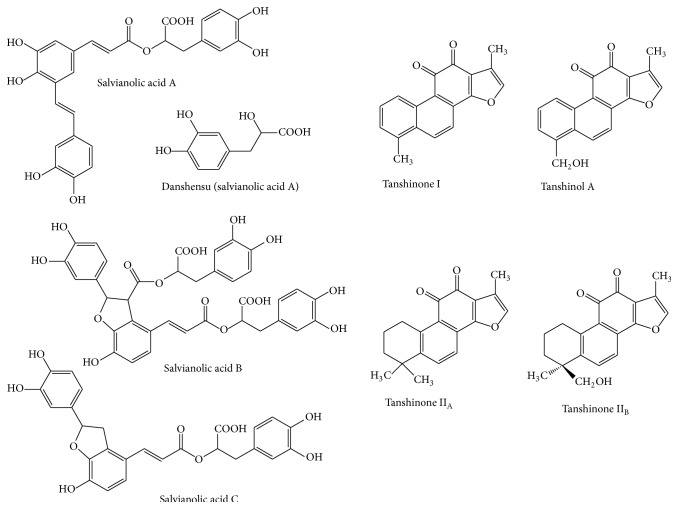
Structures of major constituents in Danshen (danshensu, salvianolic acids B and C; tanshinones Ι, *ΙΙ*_A_, and *ΙΙ*_B_, and tanshinol A).

Until now, more than 70 compounds have been isolated and structurally identified from Danshen with various concentrations. The major components reported from Danshen are hydrophilic depside derivatives (e.g., danshensu, salvianolic acids A–C, E–G, caffeic acid, and ferulic acid) and lipophilic diterpenoids (e.g., tanshinones Ι, *ΙΙ*_A_, and *ΙΙ*_B_, tanshinol A, and tanshindiols A and B). Some of the structures are given in Figure 2 [71].

In TCM, Danshen is characterized as a common hemorheological drug with the following functions: (1) to promote blood flow in menstruation, (2) to remove blood stasis, (3) to reduce pain, (4) to resolve mental uneasiness and restlessness, (5) to nourish the blood, and (6) to tranquilize the mind. Based on modern investigations, the most important and frequent clinical application of Danshen is in the treatment of coronary heart disease, like angina pectoris, coronary artery spasm, myocardial infarction, and other conditions [72]. In addition, Danshen is used to treat cerebrovascular disease, hepatitis, hepatocirrhosis, hypertension dysmenorrhea, and osteoporosis [73].

#### 4.1.2. Danshen for SCI Treatment

In recent years, Danshen has attracted increased attention in SCI treatment and is mostly employed as an intervention approach in the form of herbal extract or Chinese medicine injection. Behavioral scores (i.e., tilt board test) and histopathological changes confirmed the improvement in rat motor functions (*P* < 0.01), while bleeding and edema in the damage zone were significantly reduced after administration of Danshen (2.67 g/kg/d, i.p.) for 7 and 14 days, respectively [74].

*(1) Hemorheology Changes*. Considering the properties of promoting blood circulation and relieving blood stasis, it was reasonable that Danshen could improve microcirculation and increase blood flow of the injured spinal cord tissues, inhibit platelet aggregation, and reduce release of TXA_2_. The upregulation of hemodynamic indices of *η*b, Fib, and RAI induced by SCI was significantly reversed by administration of Danshen (6.0 g/kg, i.p., *P* < 0.01) [75].

*(2) Antioxidant and Anti-Inflammatory Effects*. Administration of Danshen injection (3 mg/kg) reversed the increase of MDA and decrease of SOD levels in white tissue of spinal cord after acute SCI (*P* < 0.01). By contrast, Danshen downregulated the increase of NO level in serum and spinal cord tissues (*P* < 0.05) [76].

Both immunohistochemistry and Western blot assays demonstrated that the increase of NF-*κ*B expression induced by SCI was ameliorated by Danshen injection (9 g/kg, twice per day, *P* < 0.05) [77].

*(3) Neuroprotective Effects*. Danshen injection (2.67 g/kg/d) could significantly increase glial cell line-derived neurotrophic factor (GDNF) (1 d after SCI), choline acetyltransferase (ChAT) (3 d after SCI), synaptophysin (7 d after SCI), synapsin Ι (7 d after SCI), and synaptic adhesion protein Ι (syt Ι) (7 d after SCI) in gray matter of spinal cord after acute SCI (*P* < 0.01) [74]. Therefore, the mechanisms of Danshen on SCI treatment could be attributed to increasing the activity of ChAT in attempt to restore the motor function of spinal cord gray matter and increase the activity of associated proteins in the synapse to promote the transfer of nerve impulses.

The mRNA expression and level of myelin basic protein (MBP) in the cytoplasm of oligodendroglia were also continuously upregulated following multiple administration of Danshen injection (50 mg/kg, twice per day) after SCI. This was important in promoting the regeneration of myelin and the recovery of neurological function [78].

### 4.2. Ginkgo

#### 4.2.1. Source, Chemical Constituents, and Pharmacology of Ginkgo

*Ginkgo biloba* L. is well-known globally although grown mainly in China and Japan. In China, the dried leaf of* Ginkgo biloba* L. is used as a medicine and is named as Ginkgo Folium [22]. In Western countries, medical interest in Ginkgo has grown dramatically since the 1980s, and extracts from* Ginkgo biloba* leaves (EGb) are one of the most commonly used herbal medicinal products in Europe and in the US today [79]. The extract taken most is the standardized extract EGb761® [80].

**Figure 3:**
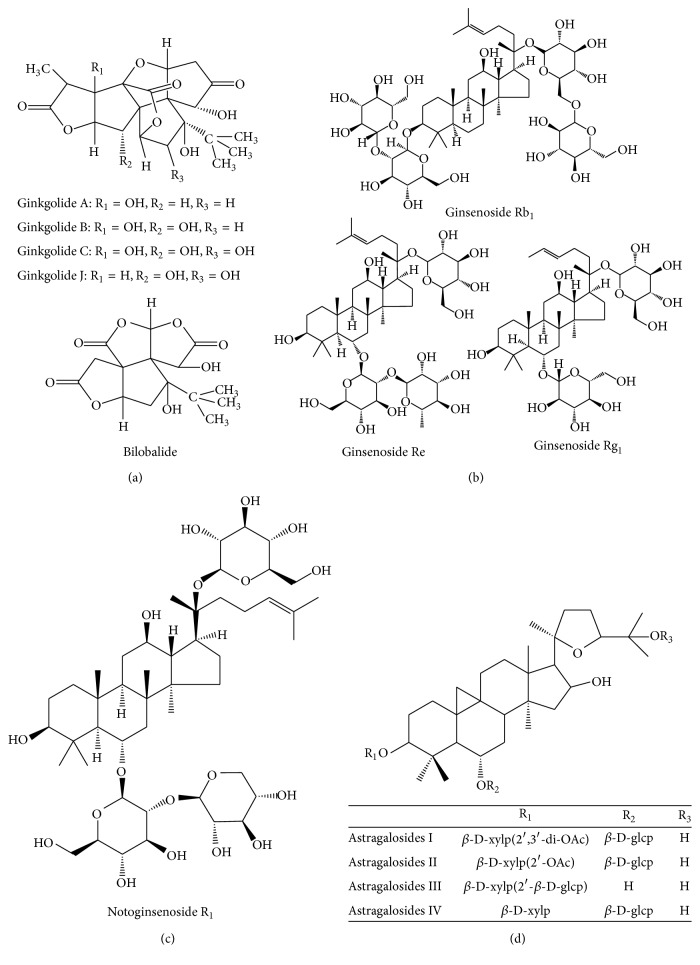
Structures of major constituents in Ginkgo, Ginseng, Notoginseng, and Astragali Radix ((a) structures of ginkgolides A, B, C, and J and bilobalide in Ginkgo; (b) structures of ginsenosides Rb_1_, Re, and Rg_1_ in Ginseng; (c) structures of notoginsenoside R_1_ in Notoginseng; (d) structures of astragalosides I–IV in Astragali Radix).

EGb has been well investigated chemically for various classes of constituents. It is reported to contain a number of secondary metabolites including terpenoids, flavonoids, polyphenols, allyl phenols, organic acids, carbohydrates, fatty acids and lipids, inorganic salts, and amino acids. However terpene trilactones (e.g., ginkgolides A, B, C, and J and bilobalide) (Figure 3(a)) and flavonoid glycosides (e.g., quercetin, kaempferol, and isorhamnetin) are considered the main bioactive constituents [81].

The therapeutic indications of EGb include chest impediment, heart pain, stroke, hemiplegia, and dysphasia due to blockage of the meridians by stagnated blood and angina pectoris of the stable type in coronary heart disease and cerebral infarction with the above noted symptoms [22]. Nowadays, EGb is widely used for diseases like cerebral ischemia, cardiovascular disease, Alzheimer's disease, dementia, and memory loss [82].

#### 4.2.2. EGb for SCI Treatment

Although EGb is not regularly used to treat SCI in Western countries and China, behavioral scores (i.e., tilt board test and BBB scores) and histopathological changes have confirmed the improvement in rat hindlimb motor functions (*P* < 0.01) [83]. HE staining results showed that rats given EGb (17.5 mg/kg/d or 25 mg/kg/d) had fewer incidences of hemorrhage, edema, necrosis, axonal demyelination, swelling of nerve cells, infiltration of inflammatory cells, and astroglial responses in spinal cord as compared with that of the control group [84, 85]. In addition, several studies suggested antioxidant and antiapoptosis effects* in vivo*, providing a possible alternative mechanism for improvement of SCI symptoms [86].

*(1) Antioxidant Effects*. Intraperitoneal injection of EGb (100 mg/kg/d) could significantly upregulate SOD levels, while downregulating MDA and NO levels (*P* < 0.05) in the injured spinal cord after SCI [87, 88].

*(2) Antiapoptosis Effects*. The presence of apoptotic cells, Bcl-2 and Bax expression, caspase-3 and caspase-9 expression, and iNOS were upregulated after SCI in the injured spinal cord (*P* < 0.01). EGb (17.5 mg/kg/d) treatment significantly increased the ratio of apoptosis cells, Bcl-2 expression, caspase-3, caspase-9, and iNOS levels (*P* < 0.05), while it decreased Bax expression (*P* < 0.01) in anterior horn motor neurons of the spinal cord [84, 85, 89].

#### 4.2.3. Active Constituents in Ginkgo for SCI Treatment

In recent years, investigators have focused attention from EGb to terpene trilactones in the field of SCI treatment, and ginkgolides A and B are two active constituents that have attracted the most attention.

The combined behavioral and BBB scores have confirmed an improvement to the recovery of muscular function and limb coherence after intraperitoneal injection of ginkgolide A (10 mg/kg/d) and ginkgolide B (2 mg/kg/d or 4 mg/kg/d), respectively [90, 91]. Ginkgolide B (2 mg/kg/d) could also improve hemorrhage, edema, necrosis, and inflammatory cell infiltrates in injured spinal cord [92].

*(1) Antiapoptosis Effects*. Ginkgolide B (2 mg/kg/d) could significantly reverse the increase of apoptosis rates (*P* < 0.05), caspase-3 expression in injured spinal cord nerve cells (*P* < 0.01), and caspase-3 p20 immunostaining positive cells in the penumbra areas (*P* < 0.01) at 3, 7, and 14 days after acute SCI [92]. Another report indicated that the underlying mechanism of Ginkgolide B (4 mg/kg/d) protection of rats against acute SCI may be related to inhibition of the JAK/STAT signaling pathway (*P* < 0.05), improvement of the Bcl-2/Bax ratio (*P* < 0.05), and decreases of caspase-3 gene and protein expression (*P* < 0.05) [91].

*(2) Neuroprotective Effects*. SP immunostaining indicated that continuous intraperitoneal injection of ginkgolide B (2 mg/kg/d) could significantly reverse the upregulation of ED-1 positive cells, S100*β*-positive cells, and GFAP-positive cells numbers 3 d after SCI (*P* < 0.01). Meanwhile, the downregulation of MBP-positive cells in the injured spinal cord region (*P* < 0.01) was also reversed by continuous administration of ginkgolide B (2 mg/kg/d) for 3 d after SCI. These results indicated that ginkgolide B could efficiently decrease hyperplasia and overgrowth of astrocytes, prevent the formation of glial scar, and decrease the accumulation of macrophages and activation of microglia, as well as preventing demyelination of axons and promoting regeneration of axons to some extent [93].

### 4.3. Ginseng

#### 4.3.1. Source, Chemical Constituents, and Pharmacology of Ginseng

Ginseng is the dried root and rhizome of* Panax ginseng* C. A. Mey. in genus* Panax* of Araliaceae family and is also known as Ginseng Radix et Rhizoma [22]. Although it is a traditional herbal medicine in oriental countries, especially China, Korea, and Japan, Ginseng has been widely used all over the world. For its promising healing and restorative properties, it has occupied a prominent position in the list of best-selling natural products in the world.

Since the first isolation of six ginsenosides derived from Ginseng in the 1960s [94], many ginsenosides have been isolated and identified. Among various ginsenosides, Rb_1_, Rg_1_, Rg_3_, Re, and Rd are the most frequently studied variants, and the structures of some typical constituents are given in Figure 3(b). Many pharmacological activities of Ginseng extracts have been discovered since the 1950s, and most of them are attributed to ginsenosides, which include antioxidant, anti-inflammatory, antidiabetic, anticancer, anti-ischemic, antiarrhythmic, antihypertensive, inhibiting platelet aggregation, adjusting lipid profiles, and improving aging [95].

#### 4.3.2. Ginsenoside for SCI Treatment

Although ginsenosides are not used to treat SCI regularly, its potential therapeutic effect in SCI treatment has been confirmed by behavioral scores (i.e., CBS scores) and histopathological changes. Both EM and HE staining showed that administration of ginsenosides (3 g/kg/d or 100 mg/kg/d) could improve the hemorrhage and edema seen in the injured spinal cord, as well as tissue necrosis, vacuolar degeneration of neurons, pyknosis and dissolution of the nucleus, disappearance of the Nissl body in the gray matter, infiltration of inflammatory cells, nerve fiber fractures, and axonal demyelination in the white matter [96, 97]. Ginsenosides protect spinal cord neurons from both oxidative stress and apoptosis* in vivo* and* in vitro*, which may be two of the major mechanisms of SCI.

*(1) Antioxidant Effects*. Intraperitoneal injection of ginsenosides (3 g/kg/d) could significantly upregulate SOD and GSH levels (*P* < 0.05), while downregulating MDA level (*P* < 0.05) and Ca^2+^ influx (*P* < 0.01) in the injured spinal cord after SCI [96].

In the oxidative stress model, spinal cord neurons were treated with 30 mM H_2_O_2_ for 24 h. Rb_1_ (≥20 mM) and Rg_1_ (≥20 mM) could significantly reduce neuronal death by approximately 52–57% (*P* < 0.01), although the protective ability against oxidative damage was limited.

In the excitotoxic model, spinal cord neurons were treated with 500 mM glutamate for 1 h (or 100 mM kainic acid for 24 h). A 20 mM concentration of Rb_1_ and 40 mM Rg_1_ appeared to be sufficient to provide full protection, as measured by both direct cell counts and neuron-specific enolase (NSE) ELISA (*P* < 0.01) [98].

*(2) Antiapoptosis Effects*. Intraperitoneal injection of ginsenosides (100 mg/kg/d) upregulated the expression of Bcl-2 protein and the ratio of Bcl-2/Bax, while downregulating the expression of Bax, caspase-3 protein, and apoptotic cell numbers (*P* < 0.01), as measured by TUNEL, immunohistochemistry, and Western blot assays [97, 99].

### 4.4. Notoginseng

#### 4.4.1. Source, Chemical Constituents, and Pharmacology of Notoginseng

*Panax notoginseng* (Burk.) F. H. Chen (Araliaceae) is a Chinese medicinal herb, distributed throughout the southwest of China, Burma, and Nepal. According to the Chinese Pharmacopoeia (2010 edition), the dried root and rhizome of* Panax notoginseng *are used as medicines, which are given the name Notoginseng Radix et Rhizoma [22].

There are various chemical constituents in Notoginseng, including ginsenosides, notoginsenosides, flavonoids, volatile oils, amino acids, and polysaccharides. Extensive chemical studies on this herb have shown that dammarane-type saponins are the main bioactive components [100]. The* Panax notoginseng* Saponins (PNS) contain protopanaxadiol glucosides (e.g., ginsenosides Rb_1_ and Rd), protopanaxatriol glucosides (e.g., ginsenosides Rg_1_ and Re), and notoginsenosides (e.g., notoginsenoside R_1_) (Figure 3(c)), which account for 12% of the root [101].

In addition, Notoginseng has various pharmacological actions and is traditionally used in the treatment of cardiovascular diseases, inflammation, trauma, and hemorrhage. It is also reported to have antihypertensive, antithrombotic, antiatherosclerotic, hemostatic, antitumour, neuroprotective, immunological adjuvant, and hypoglycaemic activities [102].

#### 4.4.2. PNS for SCI Treatment

As the similarity in chemical constituents between Ginseng and Notoginseng, it is reasonable to suspect that PNS is used in SCI treatment. Behavioral scores (i.e., tilt board test and BBB scores) have confirmed improvement in rat hindlimb motor functions (*P* < 0.01). EM showed that neurons shrank and exhibited abnormal morphology. Following the treatment of PNS (30 mg/kg/d), the ultrastructure of the neurons demonstrated a much clearer morphology, including an evenly distributed chromatin, integrative nuclear membranes with decreased introcession, and intact granular ER and mitochondria [103].

*(1) Anti-Inflammatory and Neuroprotective Effects*. Both immunohistochemical and Western blot assays indicated that PNS (30 mg/kg/d) could exert anti-inflammatory effects against SCI by reducing the expression of IL-1*β* and TNF-*α*, as well as increasing the expression of IL-10 (*P* < 0.01) [103]. Besides, AQP4 in the central ependymal cells and the gliocytes that surround the blood vessels, as well as GFAP expression in the gray matter, was significantly increased after SCI (*P* < 0.01). After PNS treatment (30 mg/kg/d or 20 mg/kg/d), AQP4 and GFAP staining was significantly reduced (*P* < 0.01), which was similar to the effect of MP therapy (30 mg/kg/d) in the positive control group (*P* > 0.05) [103, 104].

*(2) Antiapoptosis Effects*. Intraperitoneal injection of PNS (30 mg/kg/d) downregulated the increase of expression of two apoptosis-related proteins Fas and FasL in the injured spinal cord after acute SCI, as quantified by immunohistochemical and Western blot assays [103, 105].

### 4.5. Astragali Radix

#### 4.5.1. Source, Chemical Constituents, and Pharmacology of Astragali Radix

Astragali Radixis a perennial herbaceous plant of the Leguminosae family, which is widely distributed throughout the temperate regions of the world. It is derived from the dried root of* Astragalus membranaceus* (Fisch.) Bge. var.* mongholicus* (Bge.) Hsiao or* Astragalus membranaceus* (Fisch.) Bge. [22] and is one of the most popular health-promoting herbal medicines commonly used in China.

The compounds contained in Astragali Radix have been isolated and identified as polysaccharides (APS), triterpene saponins (e.g., astragalosides I–IV, AST I–IV), flavonoids, amino acids, alkaloids, and trace elements [106]. Up to now, various biological activities of these compounds or Astragali Radix extract have been investigated and reported, such as immunomodulatory, antioxidant, anti-inflammatory, antitumour, antidiabetic, antiviral, cardioprotective, antihyperglycemic, antiatherosclerotic, and hepatoprotective effects [107].

The polysaccharides and triterpene saponins have been identified as the major active ingredients responsible for the bioactivities. The structures of AST I–IV are given in Figure 3(d).

#### 4.5.2. Astragali Radix for SCI Treatment

In recent years, Astragali Radix has emerged as a potential therapeutic drug in the treatment of SCI and is mostly used as a form of herbal extract or Chinese medicine injection.

Behavioral scores (i.e., BBB scores, CBS scores, and tilt board test) and changes in somatosensory evoked potentials (SEP) have confirmed the improvement to rat motor functions (*P* < 0.01). HE staining showed that bleeding and edema were significantly reduced in the damage zone, with the necrotic area decreasing (*P* < 0.01), after intraperitoneal injection of Astragali Radix for 21 d (4 g/kg/d or 8 g/kg/d) [108, 109].

Several studies have indicated that Astragali Radix has both antioxidant and neuroprotective effects* in vivo*, providing a possible mechanism for SCI symptoms improvement. Intraperitoneal injection of Astragali Radix (8 g/kg/d or 4 g/kg/d) could significantly increase the downregulation of SOD, while decreasing any enhanced levels of MDA and GFAP expression (*P* < 0.01) in the injured spinal cord of SCI rats [110].

### 4.6. Summary

The pharmacological intervention mechanism of all five Chinese herbs in SCI treatment is summarized in Table 2. Besides the five herbs mentioned above, there are also some classic compound prescriptions currently used in SCI treatment, which are mainly Buyang Huanwu decoction and Zibu Piyin recipe [111–113]. It is worth noting that most of the related references of herbs and compound prescriptions are published in Chinese Journal. It indicates that researches of these herbs and compound prescriptions in prevention and treatment of SCI are still in a preliminary stage, and more systematic and thorough researches need to be done.

Compared with natural compounds used in the treatment of SCI, Chinese herbs have their own inherent advantages and disadvantages. On the one hand, similar “Drug-Drug Interaction” (DDI) would occur between the numerous compounds in the herb. Because of the enormous quantity and differential chemical properties of these compounds, it is too complicated to attempt clarification of the adverse reactions resulting from DDI, not to mention predicting and preventing such effects. On the other hand, multiconstituents of Chinese herbs have the superiority of synergistic effect and multitarget action, which can only be achieved by coadministration of several compounds in Western medicine. However, in order to give full play to superiority of Chinese herbs, more work needs to be done to explore their possible therapeutic mechanism.

## 5. Final Remarks

Spinal cord injury remains a frequent and devastating problem in modern society. Although there are no fully restorative treatments, in part because of the extremely complicated pathophysiologic mechanisms involved in SCI, various tissue engineering and cellular and molecular therapies have been tested in animal models. Many of these have reached, or are approaching, the clinical trials phase. However, none of them has been proven successfully in treating SCI patients to date. As an important supplementary treatment for SCI, TCM may provide benefits in the therapy or repair of the injured spinal cord, which has potential to replace the use of nonsteroidal anti-inflammatory drugs, neurotrophic factors, or even MP.

It is now of general consensus that successful functional recovery will not simply rely on a single therapeutic approach. Future clinical approaches will become increasingly diversified and multimodal and will likely include a combination of multiple strategies. In this context, TCM is bound to attract increased attention in the field of SCI treatment.

## Supplementary Material

Pathophysiological events occurring after SCI include acute, subacute, and chronic phases, while complex mechanisms are comprised. The routing clinical treatment of SCI is surgical procedures combined with methylprednisolone. Various novel strategies for SCI repair have emerged, including cell therapy, molecular therapy, and tissue engineering. Traditional Chinese Medicine is effective in SCI treatment and receives considerable research focus. The representative natural ingredients include curcumin, resveratrol, epigallocatechin gallate, ligustrazine, quercitrin, and puerarin. Commonly used herbs include Danshen, ginkgo, ginseng, notoginseng, and Astragali Radix.

## Figures and Tables

**Table 1:** Sources, structures, doses, and mechanisms of six natural compounds in SCI treatment.

Name	Source	Structure	Dose	Mechanism
Curcumin	Dried rhizome of *Curcuma longa *L. in Zingiberaceae; Curcumae Longae Rhizoma	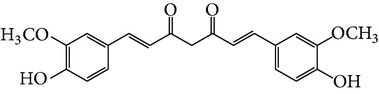	(1) *In vivo*: 100 mg/kg, i.p.; 200 mg/kg/day, p.o. (2) *In vitro*: 1 *μ*M for 24 h or 7 days	(1) Antioxidant (SOD, MDA, GSH/GSSH, GSH-PX, and CAT) (2) Anti-inflammatory (NF-*κ*B, TNF-*α*, IL-1*β*, IL-6, RANTES, and Nrf2/ARE pathway) (3) Neuroprotective effect (GFAP, NSE) (4) Antiapoptosis effect (caspase-3 and caspase-7)

Resveratrol	*Polygonum cuspidatum*, red wine, red grape skins, berries such as blueberries, peanuts, and other nuts	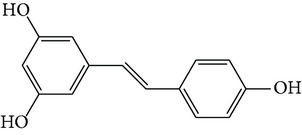	*In vivo*: 100 *μ*g/kg, i.v.; 10 mg/kg, i.v. (New Zealand white rabbit); 20 mg/kg, i.p.; 50 mg/kg, i.p.; 60 mg/kg, i.p.; 100 mg/kg, i.p.; 200 mg/kg, i.p.	(1) Antioxidant (MDA, SOD, XO, NO, and GSH) (2) Antiapoptosis (Bax, Bcl-2, caspase-3, PI3K/Akt pathway, and MAPKs signaling pathways)

EGCG	Dried fresh leaves of the plant *Camellia sinensis* L. Ktze. in Theaceae	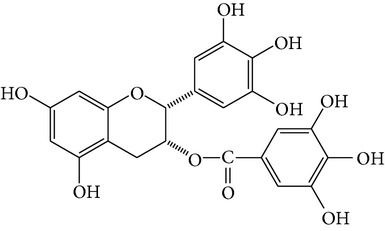	*In vivo*: 50 mg/kg, i.p.; 100 mg/kg, i.p.	(1) Antioxidant (O^2−^, SOD, and MDA) (2) Anti-inflammatory (IL-1*β*, TNF-*α*, and ICAM-1) (3) Antiapoptosis (Bax, Bcl-2, and Bcl-2/Bax ratio) (4) Neuroprotective effect (NT-3, BDNF, AQP4, and GFAP)

Ligustrazine	Dried rhizome of *Ligusticum chuanxiong* Hort. in Umbelliferae	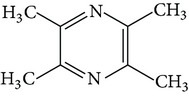	*In vivo*: 30 mg/kg, i.p.	(1) Anti-inflammatory (IL-1*β*, TNF-*α*, IL-10, and NF-*κ*B) (2) Antioxidant (MDA, SOD) (3) Antiapoptosis (Bcl-2, Bax, caspase-3, and PI3K/Akt pathway)

Quercetin	Apples, onions, citrus fruits, berries, red grapes, red wine, and broccoli	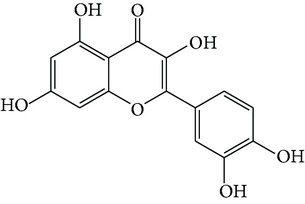	*In vivo*: 0.2 mg/kg/d, i.p.; 20 mg/kg twice daily, i.p.	(1) Antioxidant (MDA, SOD, GSH, NO, and MPO) (2) Anti-inflammatory (TNF-*α*, IL-1*β*, and IL-6) (3) Antiapoptosis (caspase-3, p38MAPK signaling pathway)

Puerarin	Dried root of *Pueraria lobata* (Willd.) Ohwi in Leguminosae	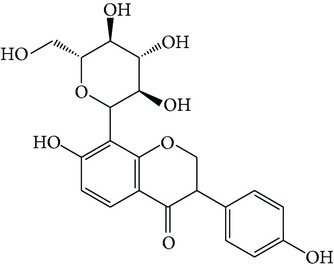	50 mg/kg, i.p.	Neuroprotective activity (p35, p25, glutamate, Trx-1, and Trx-2)

Animals used in experiments are rats without special explanation.

**Table 2:** Sources, major components, dosage form, and mechanisms of five Chinese herbs in SCI treatment.

Name	Picture	Source	Major components	Dosage form	Mechanism
Danshen (Salviae Miltiorrhizae Radix et Rhizoma)	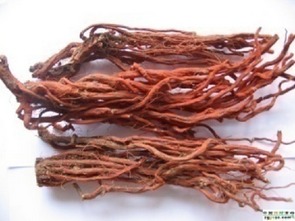	Dried root and rhizome of *Salvia miltiorrhiza* Bge. in genus *Salvia* of mint family	Hydrophilic depsides derivatives (danshensu, salvianolic acids A–C, E–G, caffeic acid, ferulic acid, etc.) and lipophilic diterpenoids (tanshinones Ι, ΙΙ_A_, and ΙΙ_B_, tanshinol A, tanshindiols A, B, etc.)	(1) Herb extract (2) Chinese medicine injection	(1) Hemorheology change (blood flow, platelet aggregation, TXA_2_, *η*b, Fib, and RAI) (2) Antioxidant (MDA, SOD, and NO) (3) Anti-inflammatory (NF-*κ*B) (4) Neuroprotective effect (GDNF, ChAT, synapsin Ι, syt Ι, and MBP) (5) Antiapoptosis (apoptotic cell index, iNOS expression)

Ginkgo (Ginkgo Folium)	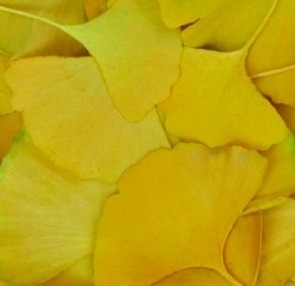	Leaves of *Ginkgo biloba* L. in Ginkgoaceae	Terpenoids trilactones (ginkgolides A, B, C, and J and bilobalide), flavonoid glycosides (quercetin, kaempferol, and isorhamnetin), polyphenols, allyl phenols, organic acids, carbohydrates, fatty acids and lipids, inorganic salts, and amino acids	(1) Extracts from *Ginkgo biloba* leaves (EGb) (2) Active constituents: ginkgolides A and B	*EGb*: (1) Antioxidant (SOD, MDA, and NO) (2) Antiapoptosis (iNOS, ratio of apoptotic cells, caspase-3, Bcl-2, and Bax) *Ginkgolides A and B*: (1) Antiapoptosis (Bcl-2/Bax ratio, caspase-3, and JAK/STAT signaling pathway) (2) Neuroprotective effect (ED-1 positive cells, S100*β*-positive cells, GFAP-positive cells, and MBP-positive cells)

Ginseng (Ginseng Radix et Rhizoma)	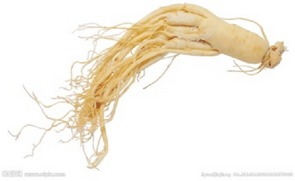	Dried root and rhizome of *Panax ginseng* C. A. Mey. in genus *Panax* of Araliaceae family	Ginsenosides (Rb_1_, Rg_1_, Rg_3_, Re, Rd, etc.)	(1) Herb extract (2) Chinese medicine injection	(1) Antioxidant (SOD, GSH, MDA, Ca^2+^ influx, and NSE) (2) Antiapoptosis (apoptotic cells numbers, Bcl-2, Bax, and caspase-3)

Notoginseng (Notoginseng Radix et Rhizoma)	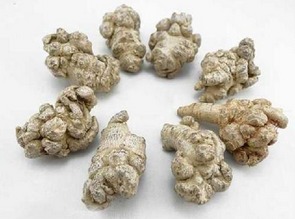	Dried root and rhizome of *Panax notoginseng* (Burk.) F. H. Chen in Araliaceae	Ginsenosides (ginsenosides Rb_1_, Rd, Rg_1_, and Re), notoginsenosides (notoginsenoside R_1_), flavonoids, volatile oils, amino acids, and polysaccharide	Herb extract: *Panax notoginseng* Saponins (PNS)	(1) Anti-inflammatory (IL-1*β*, TNF-*α*, and IL-10) (2) Neuroprotective effect (AQP4, GFAP) (3) Antiapoptosis (Fas, FasL)

Astragali Radix	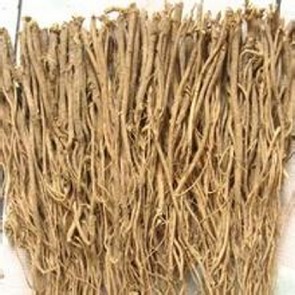	Dried root of *Astragalus membranaceus* (Fisch.) Bge. var. *mongholicus* (Bge.) Hsiao or *Astragalus membranaceus* (Fisch.) Bge.	Polysaccharides (APS), triterpene saponins (astragalosides I–IV, AST I–IV), flavonoids, amino acids, alkaloids, and trace elements	Astragali Radix injection	(1) Antioxidant (SOD, MDA) (2) Neuroprotective effect (GFAP)
