# Supplementary figures and images for: Therapeutic Effects of Traditional Chinese Medicine on Spinal Cord Injury: A Promising Supplementary Treatment in Future
Source: Evid Based Complement Alternat Med. 2016 Mar 28;2016:8958721. doi: 10.1155/2016/8958721 (PMC4826935; doi:10.1155/2016/8958721)

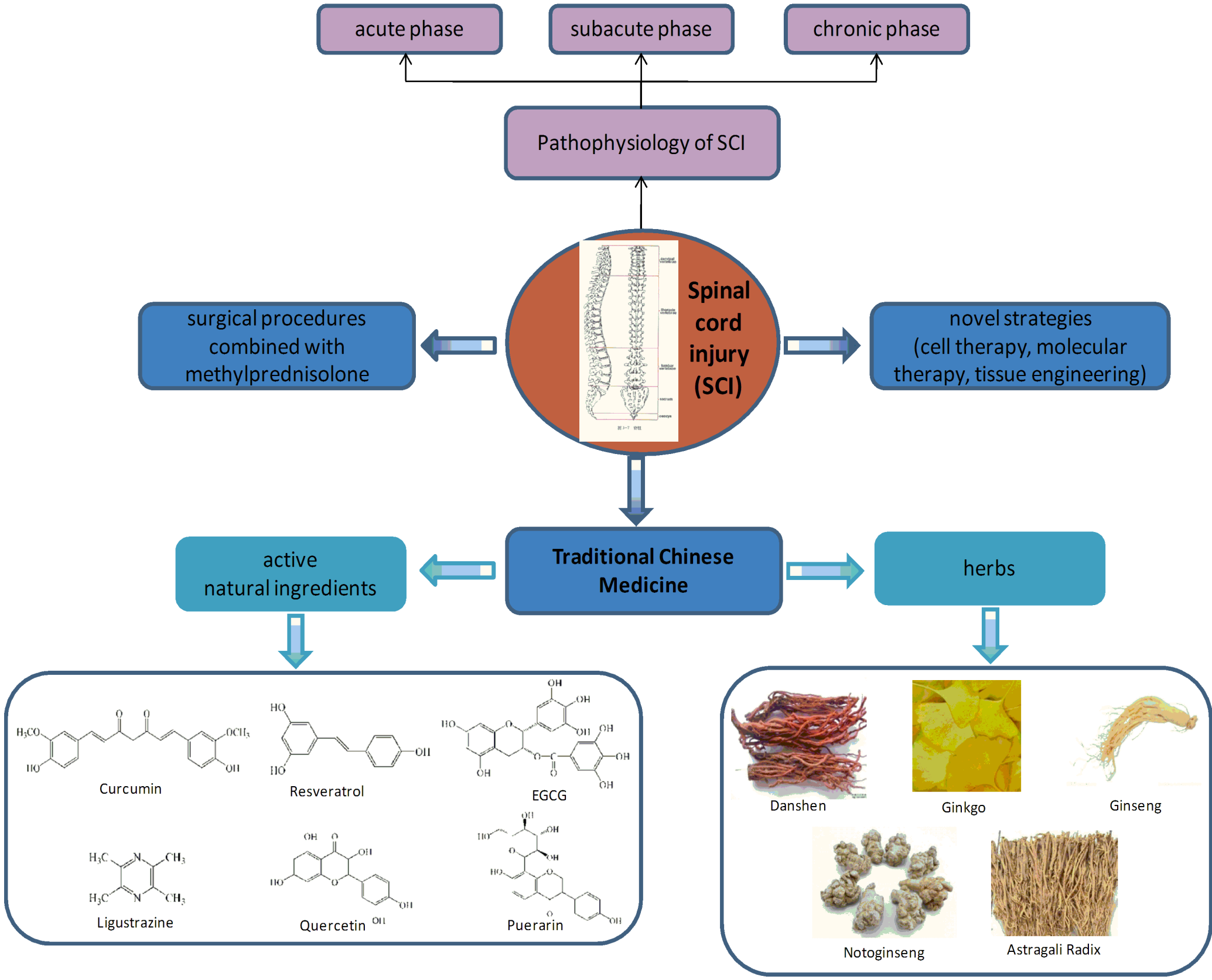

Supplement: Supplementary file 1 — Pathophysiological events occurring after SCI include acute, subacute, and chronic phases, while complex mechanisms are comprised. The routing clinical treatment of SCI is surgical procedures combined with methylprednisolone. Various novel strategies for SCI repair have emerged, including cell therapy, molecular therapy, and tissue engineering. Traditional Chinese Medicine is effective in SCI treatment and receives considerable research focus. The representative natural ingredients include curcumin, resveratrol, epigallocatechin gallate, ligustrazine, quercitrin, and puerarin. Commonly used herbs include Danshen, ginkgo, ginseng, notoginseng, and Astragali Radix. [file 8958721.f1.pdf]
